# Testimonial Dinner to Dr. J. M. Bodine of Louisville

**Published:** 1910-12

**Authors:** 


### Testimonial Dinner to Dr. J. M. Bodine of Louisville.

FOR forty-five years Dr. James Morrison Bodine, as dean and president of the faculty, has presided over the destinies of the University of Louisville Medical Department. Now entering his eightieth year he continues to devote his energies to the cause of medical education. His many friends and pupils scattered throughout the country have deemed this an appropriate occasion to show their appreciation of his devotion and their affection for him personally.

To this end it has been decided to give him a testimonial dinner December 16, 1910, at the Seelbach Hotel in Louisville. Those friends whom the Committee has been unable to reach by invitation are cordially invited to attend and are requested to signify such intention at once by writing to the chairman of the committee.

The cost of each plate will be ten dollars ($10.00).

Signed:

Lewis S. McMurtry,
J. M. Mathews,
Ap Morgan Vance,
J. M. Ray,
Thos. L. Butler,
I. N. Bloom, *chairman.*

---

Dr. W. A. Evans, Chicago's Health Commissioner and leading phrase maker (*The Tribune*) is out with a new batch of "healthograms." "Too much fresh air is just enough," says Dr. Evans, and following this up he declares that "the biggest bedroom in the world is not big enough to sleep in if the windows are shut." The *Evening Post* says that under Dr. Evans's guidance "Chicagoans are rapidly learning how to be healthy. They are learning the importance of fresh air, are finding out how to avoid disease and are seeing it demonstrated that people can live in Chicago and have good health, despite city conditions."

AUTHORITY has been granted to Superintendent of Education Emerson to start what is called an open-air school in district No. 23, on Delevan Avenue, Buffalo, for children recommended by the medical school inspectors and passed on by an officer of the health department. Not more than twenty-five pupils will be taken in this first experimental class. The teacher in charge is to receive a salary of $950 a year.

---

A SERIES of health talks for the public schools of Buffalo has been arranged and the program has been completed by Dr. Schaefer, head of the publicity bureau of the health department. The city has been divided into five parts, the assignments and topics being Division No. 1, Dr. W. C. Callanan, "Cleanliness;" Division No. 2, Dr. Edward Durney, "The Spread of Contagious Diseases;" No. 3, Dr. McClure, "The Early Recognition of the Common Contagious Diseases;" No. 4, Dr. F. W. Barrows, "Fresh Air As an Aid in Education," and No. 5, Dr. Nairn, "The Factors in Hygiene of School Children, Proper Feeding, Care of Teeth and Mouth, and Prevention of Deformities."

---

THE HUDOR WATER COMPANY, of Buffalo, manufactures a pure ginger ale, absolutely free from capsicum or any other deceptive ingredient. It has been our privilege to visit the Hudor laboratories and witness the process employed in creating for the market various waters and "soft drinks." We were impressed particularly with those in vogue in the manufacture of ginger ale. Here we observed Jamaica-grown ginger root made use of as the basic element, which is passed through the process of grinding, maceration, filtering, flavoring with pure lemon extract made from the peel before our eyes, and otherwise being made ready for use. We are interested in ginger ale because we regard it as a useful stomachic, and especially valuable for domestic use at table, or between meals if desired, in summer and in winter, indeed, in all seasons. The element of purity is important. The Hudor Water Company's product cannot be challenged. A ginger ale made from ginger root is incomparably better than "ginger" ale made from capsicum.

---

